# Global Long-Term Care Research: A Scientometric Review

**DOI:** 10.3390/ijerph16122077

**Published:** 2019-06-12

**Authors:** Liping Fu, Zhaohui Sun, Lanping He, Feng Liu, Xiaoli Jing

**Affiliations:** 1 College of Management and Economics, Tianjin University, Tianjin 300072, China; Lpf3688@126.com (L.F.); nkliufeng@126.com (F.L.); 2 Center for Social Science Survey and Data, Tianjin University, Tianjin 300072, China; 3 Department of Integrated Studies in Education, McGill University, Montreal, QC H3A 1Y2 Canada; jing.xiaoli@mail.mcgill.ca

**Keywords:** long-term care, scientometric review, CiteSpace, visualization

## Abstract

Since the early 1960s, long-term care (LTC) has attracted a broad range of attention from public health practitioners and researchers worldwide and produced a large volume of literature. We conducted a comprehensive scientometric review based on 14,019 LTC articles retrieved from the Web of Science Core Collection database from 1963 to 2018, to explore the status and trends of global LTC research. Using CiteSpace software, we conducted collaboration analysis, document co-citation analysis, and keyword co-occurrence analysis. The results showed a rapid increase in annual LTC publications, while the annual citation counts exhibited an inverted U-shaped relationship with years. The most productive LTC research institutions and authors are located primarily in North American and European countries. A simultaneous analysis of both references and keywords revealed that common LTC hot topics include dementia care, quality of care, prevalence and risk factors, mortality, and randomized controlled trial. In addition, LTC research trends have shifted from the demand side to the supply side, and from basic studies to practical applications. The new research frontiers are frailty in elderly people and dementia care. This study provides an in-depth understanding of the current state, popular themes, trends, and future directions of LTC research worldwide.

## 1. Introduction

The aging population is growing at an unprecedented rate worldwide. According to the 2017 Revision of the World Population Prospects, the global proportion of people aged 65 years and over has risen from 5.08% in 1950 to 8.29% in 2015 and is expected to reach 15.82% by 2050 [1]. Elderly people are generally at substantial risk of functional limitations and physical disability, creating a great demand for long-term care (LTC) services. Within this context, developing an effective, equitable, and sustainable LTC system to meet the escalating demand becomes increasingly important. In the last half century, LTC has been a major public health issue in nearly all industrialized countries, and a variety of LTC systems have been established based on a mix of financing sources [2,3,4]. Although these systems have grown rapidly, reflecting their popularity among the elderly, they have also faced several challenges, including skyrocketing costs and workforce shortages [5,6]. Moreover, some developing countries have started to pay attention to LTC in recent years because their demands for it are rising dramatically at a rate that exceeds that experienced by industrialized countries [7,8,9].

Given that LTC is a matter of serious concern in many countries, researchers have shown considerable interest in this subject [10,11]. Extensive LTC studies have been conducted from the perspectives of geriatrics and gerontology; nursing; public, environmental, and occupational health; health care sciences and services; infectious diseases; psychiatry; psychology; and economics, including demographic trends and health status [12,13,14], demand for and supply of LTC [3,5,15,16], LTC workforce [17,18,19], LTC expenditure and financing [2,6,9,20], and LTC reforms [4,21]. Clearly, LTC is a broad and complex research field that combines different disciplines, which to some extent has contributed to the fragmented nature of LTC research. It is thus necessary to gather the published LTC data and conduct a large-scale review of scientific studies to fully comprehend LTC research development.

Previous LTC reviews have focused mainly on specific subfields and themes. For example, Norton [16] carried out a research review on the supply of and demand for LTC. Hussein and Manthorpe [18] reviewed LTC institutional arrangements in some developed countries and explored different strategies used to resolve an LTC workforce shortage. Seitz et al. [14] presented a systematic review on the prevalence of eight common psychiatric disorders in LTC populations. Wong and Leung [22] analyzed the issues facing LTC services and reviewed their prospects, including the structure, operation, financing, and challenges. While this narrow focus helps deepen our understanding of specific facets of LTC, the resulting fragmentation of LTC research prevents us from seeing the overall picture. Additionally, a common problem in these studies is that they are qualitative reviews and prone to subjectivity. Little attention has been paid to a quantitative review of LTC research.

The present study aimed to address these gaps in the existing literature by undertaking a comprehensive and in-depth scientometric review of global LTC research, with a view to assisting researchers to better understand the LTC field. Specifically, our review is guided by three key goals: (1) to depict the current status of LTC research from the perspectives of publication and citation output; (2) to identify the major contributors to LTC research in terms of countries, institutions, and authors; and (3) to map the intellectual landscape of the LTC field based on a dual perspective of references and keywords, to reveal the research hotspots, frontiers, and trends. Our study differs from previous research in three ways: we reviewed LTC research as a whole instead of focusing on its subfields; we used a broad literature search for all relevant articles instead of focusing on the key articles from specified journals; and we used a quantitative method to objectively review LTC research, complementing earlier reviews.

## 2. Materials and Methods

### 2.1. Scientometric Analysis in CiteSpace

We employed CiteSpace version 5.1.R8 SE, a freely available Java-based software package developed by Dr. Chaomei Chen at Drexel University (Philadelphia, PA, USA), to conduct scientometric analyses [23]. Recently, CiteSpace has received increasing attention for its strength in uncovering and visualizing the structural and temporal patterns of knowledge domains through systematically generating various graphs [23,24,25,26,27,28,29,30,31]. Visual maps created by CiteSpace are composed of two elements, nodes and links, with the former representing countries, institutions, authors, cited references, keywords and so on and the latter representing the co-occurrence or co-citation relationship between nodes [25]. Nodes with large size (determined by publication or citation frequency), purple rings (determined by centrality), or red inner rings (determined by burst) are usually identified as three major types of nodes which may influence the development of a scientific research domain [26]. Similarly, a thicker link shows a stronger relationship between two nodes in a connection.

The general procedures for visualization analysis with CiteSpace are outlined as followed: (1) identify a knowledge domain; (2) collect data; (3) extract research front terms; (4) specify time slicing; (5) set up thresholds; (6) select pruning and merging approaches; (7) select the layout styles; (8) conduct visual inspection; (9) verify pivotal points; (10) reach conclusions [23]. More information on how to utilize CiteSpace for a scientometric review of a research field are available in the literature (see Chen [23], Chen et al. [25], Chen and Wu [26], Kim and Chen [27], Lee et al. [28], Song et al. [29], Zhu et al. [31], and Fang et al. [32]).

Despite the popularity of CiteSpace, to the best of our knowledge, it has not previously been used to map global LTC research. To provide a systematic review on LTC research and achieve the expected objectives, three types of scientometric techniques provided by CiteSpace were applied in this study: collaboration analysis, document co-citation analysis, and keyword co-occurrence analysis. Collaboration analysis takes authors’ names, countries of affiliation, and institutional affiliation as the units of analysis and evaluates their publication contributions and academic influences by visualizing scientific collaboration networks [26,29,32]. Document co-citation analysis provides insights into the intellectual structures of a knowledge domain and identifies the quantity and authority of references cited by publications [23,24,25,28]. In the process of this analysis, cluster views and timeline views are performed to reveal the conceptual structures and the evolution of scientific activity. Keyword co-occurrence analysis tracks the research hotspots, frontiers, and trends over time by establishing a network of co-occurring keywords that provide information about the core content of articles [27,31]. Specifically, the research frontiers and evolution trends are identified by burst detection [23]. During execution, the parameters (e.g., time slice, node type, and pruning) in CiteSpace should be properly selected in accordance with the research objectives [29].

### 2.2. Data Collection

CiteSpace data collection required two steps. First a globally representative database was selected. Our review was based on the bibliographic records obtained from the Science Citation Index Expanded (SCI-E) and Social Sciences Citation Index (SSCI) in the Thomson Reuters Web of Science Core Collection (WoSCC) database, which is considered one of the most authoritative data sources for bibliometric investigations because it contains the leading global scholarly journals and is continuously dynamically updated [29]. The second step was to design an accurate retrieval strategy. The dataset was retrieved in this study by an LTC topic search, which is an ideal retrieval mode because it can characterize the article content from the perspectives of title, abstract, and keyword. We conducted a topic search for “long-term care” articles published between 1963 (the first article related to LTC was published in this year) and 2018 (downloaded June 27, 2018). The search was limited to journal articles and resulted in the 14,019-article dataset used in the scientometric analysis. In order to examine the effectiveness of the results, we assessed the relevance of the top 100 most cited articles to LTC. The results showed that 83 articles (83%) were closely related to LTC, indicating that our retrieval strategies and search term are appropriate.

## 3. Results and discussion

### 3.1. Current Status of LTC Research

**Figure 1:**
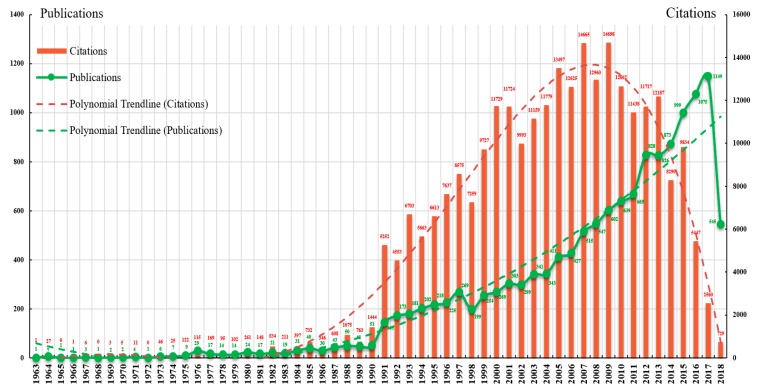
Year-wise distribution of publications and citations from 1963 to 2018.

The 14,019 LTC research articles were published in 18 different languages, although English (13,479) was the predominant language, accounting for 96.15% of the total. Among non-English languages, German (277) and French (103) were the most widely used, contributing 1.98% and 0.74%, respectively. The current status of LTC research is depicted by the year-wise distribution of publications and citations shown in Figure 1. The green points represent the number of publications per year and exhibit a noticeable upward trend. The bar graphs illustrate the annual citation counts, showing a trend that initially increased then decreased. Additionally, two trendlines were identified by fitting a polynomial to the data, as revealed by the dotted lines. Three research stages were summarized according to publications, as follows:

1. Low-speed development stage (1963–1975). The annual number of publications increased slowly in this period and never exceeded 10. There was only one article in the years 1963, 1965, 1966, 1968, and 1972. Notably, 1975 was the most productive year with nine articles. In terms of annual citation counts, the dominant position was occupied by the year 1975 with 122 citations; there were fewer than 50 citations in all other years, probably because of a limited number of publications in those years.
2. Rapid development stage (1976–1990). With a negligible amount of fluctuation, the number of publications per year increased steadily over this period. The most productive year was 1990 with 51 articles. Moreover, an increased number of citations per year were also observed over time. Articles published in 1988 and 1990 received many citations (each more than 1000), and the greatest average number of citations per article (28.31) also belonged to 1990.
3. High-speed development stage (1991–2018). Publication output increased rapidly from 145 in 1991 to 1149 in 2017, resulting in a nearly eightfold increase in the past 27 years. In this period, the polynomial trendline of publication growth showed a significant correlation between publication year and publication counts. Through curve fitting, the number of publications was estimated to reach 1217 in 2018 (1222 articles were retrieved from this year) and 1302 in 2019, indicating that LTC research may remain active for many years. Moreover, three publication bursts were found in 1991, 2012, and 2015, increasing by 94, 159, and 126 over the previous year, respectively. Note that WoS provides abstracts and keywords for articles published after 1990. This may be the reason for the strong increase in publications from 1990 to 1991. The bar graph with an inverse U-shaped trendline showed that the number of citations peaked in 2009 with a record of 14,698 and then began to gradually decline, probably as a result of the time required for the accumulated effects of new publications.

### 3.2. Major Contributors to LTC Research

LTC has become a popular research subject, with varying degrees of concern across countries, institutions, and authors. Who are the predominant contributors? How much difference do they make? What research network do they have? These questions are fundamental to identifying the major contributors to LTC research, but they have not been fully explored in the research. We attempted to answer these questions by mapping three collaboration networks.

#### 3.2.1. Country Collaboration Analysis

An analysis of collaboration among countries was conducted by focusing on the affiliation location of at least one author of each published article [29,31]. The following parameters in CiteSpace were used: (1) time slice from 1963 to 2018; (2) years per slice = 1; (3) term source = title /abstract/author keywords/keywords plus; (4) node type = country; (5) pruning = none; (6) select top 50 most cited articles per slice. After running CiteSpace, results revealed that 68 countries/ regions contributed to LTC publications. Table 1 shows the top 15 contributors, ranked by publication counts and centrality, respectively. The LTC contributions primarily originated from the US, Canada, and England. Specifically, the US ranked first by contributing 6308 (45%), followed by Canada with 1714 articles (12.23%) and England with 815 articles (5.81%). In terms of the large number of publications, it seems that these three contributors form a leading LTC research group.

**Figure 2:**
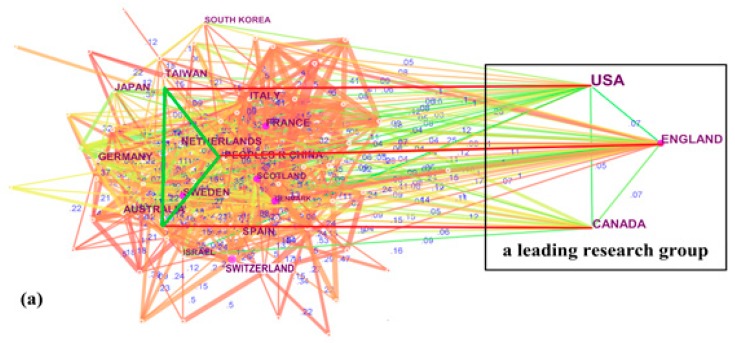

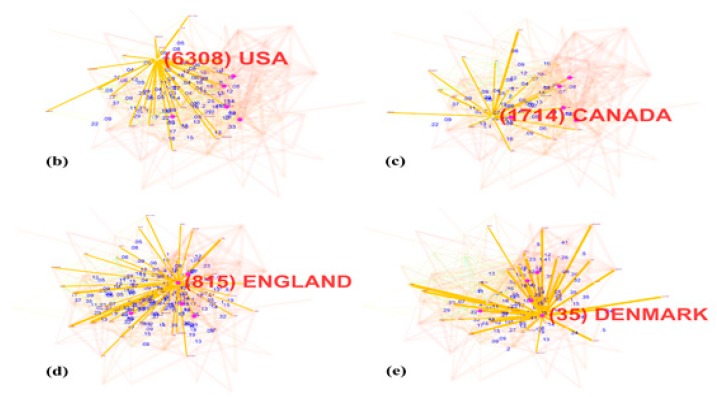
Comparison of collaboration network of contributors. (**a**) All countries/regions, (**b**) USA, (**c**) Canada, (**d**) England, (**e**) Denmark.

In Figure 2a, each node is a country or region and each link represents the relationship between two nodes. The figures indicate link-strength between the nodes, mainly ranging from 0.15 to 0.35. Among members of the leading group, the link strength of England-USA, England-Canada, and USA-Canada was 0.07, 0.07, and 0.05, respectively, showing limited collaboration. Additionally, the links between other contributors included in the network and the leading group (England-USA-Canada) were not strong, with the strength between 0.5 and 0.12. It is thus necessary to take this into account by adjusting research directions to further improve the level of collaboration among countries.

The collaborative relationships among the three group members were primarily established in the 1990s. Moreover, we found that developing countries/regions were still under-represented in the global LTC research network, although they were making great efforts to develop LTC. As shown in Table 1, China was the only developing country (Taiwan Province of China is treated as a developed region) among the top 15 most productive contributors.

Generally, nodes with high centrality (≥0.1) in the network are indicated by purple rings and connect more links. As shown in Table 1, the top six contributors in terms of centrality were England (0.16), Denmark (0.15), France (0.12), Scotland (0.12), Sweden (0.11), and Switzerland (0.1). Collaboration networks of four countries are shown in Figure 2b–e. England and Denmark, with high centrality had a dense network structure, showing that they occupied key positions on the critical paths in the network and played important roles in connecting with others. However, the limited number of publications significantly weakened Denmark’s influences. Canada, with a centrality of 0.03, had a sparse network structure, demonstrating that it did not actively participate in collaborative research activities. Its power, however, cannot be ignored because Canada was the second most productive country. Taking publications and centrality into account simultaneously, the US stands out as the most important contributor to LTC research.

#### 3.2.2. Institution Collaboration Analysis

**Figure 3:**
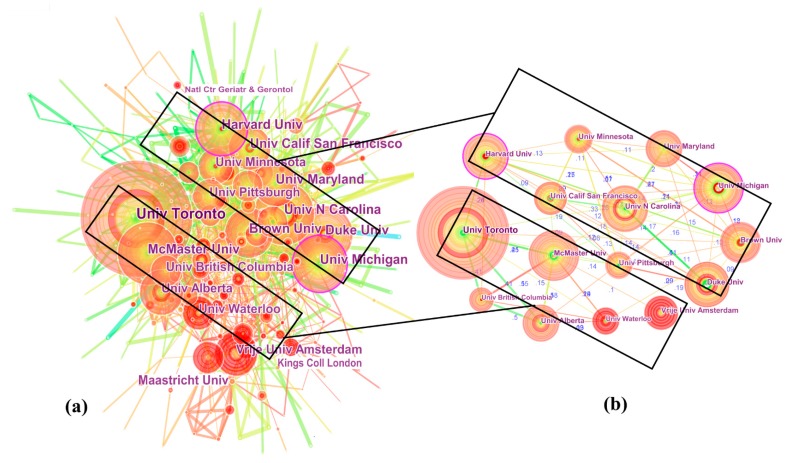
Collaboration network of institutions. (**a**) All institutions, (**b**) Top 15 most productive institutions.

In this section, the parameters in CiteSpace were kept the same, except for node type being changed from “Country” to “Institution”. To visualize the influential institutions and the collaborations between them, a network comprising 438 institutions and 1740 collaboration links is depicted in Figure 3a, showing that the top 15 most productive institutions were split into three major groups: clustering from nine American universities; five Canadian universities; and one Netherlandish university (see Table 2). The result further confirms previous findings and attests to the importance of European and North American LTC contributions [2]. In terms of publications, 15 institutions issued 3047 articles, accounting for 21.74% of the total. Specifically, UT ranked first with 406 articles (2.9%), followed by UNC (233, 1.66%) and Harvard (231, 1.65%). One prominent institution in Europe was VU with 155 articles (1.11%), ranking 12th. Moreover, we noted that Asian institutions accelerated efforts to participate in LTC research, including National Yang-Ming University (90, 0.64%) and the University of Tokyo (75, 0.54%). Apart from the universities, several research centers also played important roles in LTC research. For example, the Centers for Disease Control and Prevention (CDC) in Atlanta GA, USA ranked 19th with a total of 118 articles.

The collaboration network of 15 highly productive institutions is mapped in Figure 3b. The link strength ranged from 0.07 to 0.58 with a mean of 0.18. Research collaboration was most active between McMaster and UA (0.58), followed by McMaster-UBC (0.55) and UNC-Duke (0.51). LTC relationships were established mainly at the beginning of the 21st century, while the UNC-Duke relationship was created in the early 1990s. As for centrality, Harvard (0.14) and UM (0.12) represented the major turning points, acting as bridges linking others in different phases. Moreover, nodes with red inner rings are institutions with strong bursts, i.e., strength. In Figure 3, bursts (strength > 20) that reflected significant increases in publications over short periods of time occurred in Maastricht University (28.38, 2015–2018), King’s College London (23.13, 2014–2018), VU (21.58, 2013–2018), and the National Center for Geriatrics and Gerontology (20.95, 2015–2018), indicating that the articles from these four institutions attracted special attention over the past five years (2014–2018) and strongly contributed to LTC research development.

#### 3.2.3. Author Collaboration Analysis

**Figure 4:**
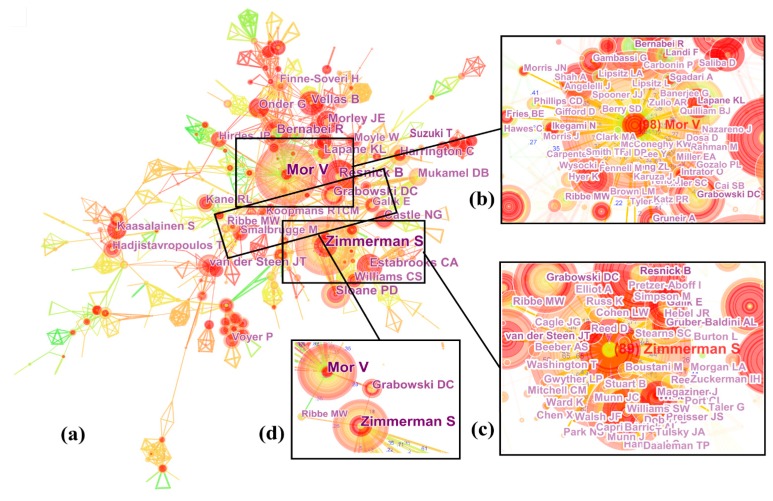
Collaboration network of authors. (**a**) All authors, (**b**) Mor, (**c**) Zimmerman, (**d**) Ribbe and Grabowski.

A scientific co-authorship network provided information on the core authors and potential collaborators and helped researchers to establish collaborative relationships. The parameters in CiteSpace were remained the same except node type being changed from “Institution” to “Author”. The distribution of authors is depicted in Figure 4a. This network consisted of 1759 nodes and 3186 collaboration links. Notably, a small group of highly productive authors contributed to a significant share of publications. For example, 2.84% of authors (top 50 authors) were responsible for 10.6% (1486) of the publications. Among all the authors, Mor was the most productive with 98 articles, followed by Zimmerman with 89 articles (see Table 3). Additionally, all authors had low centrality (<0.1), revealing that collaboration among them was insufficient. In Figure 4, bursts (strength ≥ 10), as indicated by red inner rings, occurred in Vellas (13.74, 2014–2018), Lapane (12.69, 1998–2002), Williams (11.8, 2005–2008), Van der Steen (10.83, 2011–2015), and Suzuki (10, 2015–2018). Given that bursts are often accompanied by subsequent increasing trends, research output from Vellas and Suzuki, who both had strong bursts in the last three years (2016–2018), was expected to continue to increase.

Figure 4b,c show the collaboration networks of Mor and Zimmerman, respectively. The authors tended to collaborate with a single, highly productive author, forming co-author clusters. For example, Mor was a central author of one research community, which included Grabowski, Bernabei, Lapane, etc. As compared with Figure 4c, Figure 4b depicts a much denser network structure; Mor connected more links than Zimmerman, indicating that Mor seemed more active and maybe played a more important role in LTC research. Interestingly, no direct collaborative relationship was established between the two major authors. As shown in Figure 4d, Grabowski (37, Harvard University) and Ribbe (16, VU University Medical Center) were the only two authors who made a great contribution to bridging the collaborative gap between Mor and Zimmerman.

To further identify core authors’ research contributions, we listed the top six most cited articles published by Mor and Zimmerman, respectively (see Table 3). Mor, a well-known professor in the Brown University School of Public Health, has a long history of conducting research projects on the quality of nursing home care, the determinants of hospitalization, and racial discrimination in health care treatment. During the past 30 years, Professor Mor frequently adopted an integrated research approach that combined quantitative and qualitative data analyses in program evaluations, such as nursing home resident assessment. Moreover, he is one of the authors of the congressionally mandated Minimum Data Set (MDS) for nursing home resident assessment which was designed to assess elders’ functional status and care needs. Overall, the above contributions are fully embodied in the top six most cited articles from Mor.

Similarly, Zimmerman was a Kenan Distinguished Professor at UNC-Chapel Hill and a highly representative scholar in geriatrics and gerontology. Over the last 16 years (2002–2017), she was the most productive scholar in LTC research, and has conducted groundbreaking research and intervention studies in nursing homes and assisted living communities and served as a clinician in a range of LTC settings. The six landmark articles from Zimmerman show that she has paid close attention to LTC recipients with dementia.

### 3.3. Intellectual Landscape of LTC Field

In this section, we adopted a rigorous analytical framework of intellectual landscape to identify hot topics, research frontiers, and evolution trends in LTC research based on a dual perspective of references and keywords [23,33]. Two results gained from the analysis were compared and corroborated each other; thus, this study’s findings were more comprehensive and definite than those stemming from the single perspective of references or keywords used in previous studies. The analyses were conducted as follows:

#### 3.3.1. Document Co-citation Analysis

Generally, this analysis clusters the related references into groups according to their link strengths; then, the hot research topics can be identified through analysis of the articles in each cluster. The main features of the references’ intellectual landscape used in this investigation were: highly cited references; references with high centrality; and references with strong citation bursts (i.e., burst references). The highly cited references characterize the long hotspots of a given research field [29,33]. References with high centrality are considered anchors for the evolutionary path and research root of a discipline because they are major intellectual turning points [30]. Burst references that reflect a strong citation count surge over a short period of time provide predictive indicators of research frontiers and trends [26].

**Figure 5:**
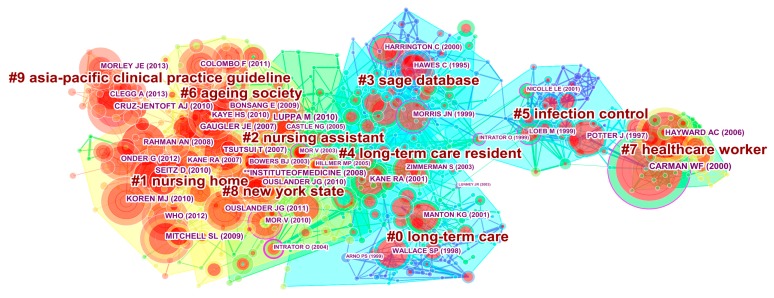
Document co-citation network in LTC research.

We conducted a co-citation analysis to identify LTC knowledge clusters. The following parameters in CiteSpace were used: (1) time slice from 2000 to 2017; (2) years per slice=1; (3) term source=title/abstract/author keywords/keywords plus; (4) node type=cited reference; (5) pruning=none; (6) select top 50 most cited articles per slice. After running CiteSpace, a total of 273,837 valid references cited in 11,115 articles over an 18-year time span were extracted, and a network consisting of 725 nodes and 2316 links was visualized. We used a short time span because of the sparse research output in the 37-year period from 1963 to 1999, and incomplete records in 2018. The network was divided into 71 clusters, which were automatically labeled by choosing title terms as the labelling source and log-likelihood ratio (LLR) as the standard algorithm. Among these, we focused on the top 10 largest clusters shown in Figure 5. Each node represents one cited reference, and each link indicates the co-citation relationship. Moreover, cluster size measured by the number of references is sequenced in descending order of the cluster numberings.

According to the three features mentioned above, the top 25 most cited references are listed in Table 4, from No. 1 to No. 25, assigned to 10 clusters. To better characterize the interrelationships among the clusters, 13 references with high centrality were presented as No. 1 and from No. 26 to No. 37. Sixteen references (i.e., No. 1, 3–5, 10, 14–16, 24, 25, 32–34, 38–40) with the strongest bursts in the group of references that started to burst at the same time can be adopted to disclose the LTC research frontiers and trends. Detailed descriptions of 40 representative references are shown in Table 4.

Cluster #0, long-term care, ranked first in cluster size, containing 87 references that were mostly published around 1998 [12,48,50,60]. In the last decades, topics related to LTC changed significantly and numerous research themes developed rapidly. Nonetheless, studies shared a common aim: to meet care needs of residents in LTC facilities. The two most active citers to this cluster were references No. 23 and No. 25. Wallace et al.’s [48] arguments have received much attention, with results suggesting that any expansion of community-based LTC should take into account older minorities’ need patterns and potential access barriers. Manton and Gu [50] assessed changes in the prevalence of chronic disability in the United States black and nonblack elderly population based on a 1999 National LTC Survey. Reference No. 35 focusing on the patterns of functional decline at the end of life showed high centrality, linking cluster #0 to clusters #4 and #5.

The second largest cluster (#1) contained 86 references focused mainly on nursing home-related topics published around 2009 [1,13,14,21,39,40,45,63]. A nursing home (NH) is defined as a facility with a domestic-styled environment that provides 24-hour functional support and care for people who require assistance with activities of daily living and who often have complex health needs and increased vulnerability [64]. There are six highly cited references in this cluster. Among them, three references had a common interest in dementia care [13,14,40]. The report from WHO [40] regarded dementia as a public health priority. Recently there has also been interest in improving quality of care for NH residents by proposing practical programs, like the NH culture-change movement and the interRAI instrument for LTC facilities [21,39,45].

Cluster #2, nursing assistant, ranked third in cluster size, including 84 references that were mostly published around 2004 [4,17,19,53,56,62]. In the NHs, nursing assistants (NAs) are responsible for a considerable amount of direct patient care and resident handling. Recent evidence has shown that the present health care workforce is small and is ill-prepared for the future surge in LTC needs, although NAs are increasingly employed to provide high-quality care for residents [19]. To address this, public health nurses were formally integrated into LTC insurance in Japan [4]. Three references with high centrality concentrated on quality indicators used to measure quality of care, and its relationship to NH profit status and NH staff turnover [17,53,56]. Obviously, quality of care has become a hot theme in LTC research.

There are other clusters worth mentioning. Cluster #3 focused on the SAGE database and its applications [49,52]. The most active citer to cluster #4 [10,57,61] was Kane [10] who wanted to bring LTC and a good quality of life closer together. Four references in cluster #5 reflected a common theme—the prevention and control of common infections in LTC facilities [46,55,58,59]. Cluster #6, ageing society, has been widely used as a context for research on NH admission [35,38], and providing and paying for LTC [5,6,47]. The two most active citers to cluster #7 examined the effects of caregiver influenza vaccination on mortality in elderly people [34,36]. Cluster #8, New York State was commonly chosen as a typical example to examine the frequency, reasons, and costs of potentially avoidable hospitalization or rehospitalization [41,44,51,54], because it has the highest countrywide Medicaid reimbursement rate and generous bed-hold policies [65]. The newly formed cluster #9 with mean publication year 2011 contained three representative references with high citation counts, which revealed a common research theme—frailty in elderly people [37,42,43].

Overall, the focus of these 10 clusters can be divided into 12 hot topics, including health status, mortality, care needs, dementia care, quality of care, formal and informal caregivers, database application, infection control, NH admission, providing and paying for LTC, potentially avoidable hospitalization or rehospitalization, and frailty in elderly people. Moreover, some analytic techniques, including randomized controlled trials and meta-analysis, received increased attention.

**Figure 6:**
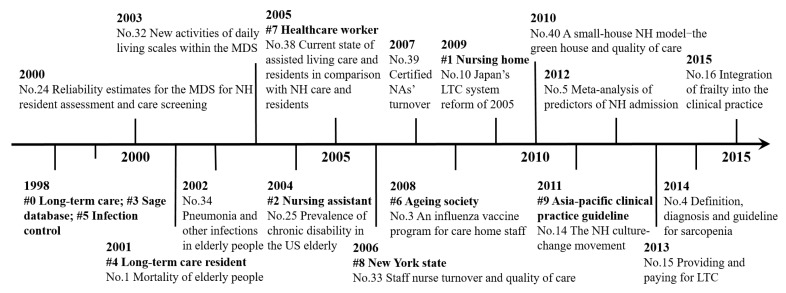
Timeline of the 10 largest clusters and 16 burst references.

The research frontiers in a certain field can be identified by references with strong citation bursts. A citation burst indicates the likelihood that the scientific community has paid or is paying special attention to the underlying contribution [25,27]. In investigating these references, we focused mainly on discussing 16 burst references (i.e., No. 1, 3–5, 10, 14–16, 24, 25, 32–34, 38–40) in Table 4. Moreover, the largest 10 emerging clusters were considered when detecting LTC research frontiers because each cluster represented a thematic concentration in the bibliographic landscape [25,33]. A timeline view of 10 clusters and 16 burst references with their respective research foci is shown in Figure 6. The LTC research trends at different times were revealed as follows: in the early stage from 2000 to 2005, research focused on health status and care needs among elderly LTC residents; in the second stage from 2006 to 2010, focus shifted to aspects of the LTC workforce, such as nurse turnover, vaccination of care home staff, and nurse training; in the third stage from 2011 to 2015, some practical programs aimed at improving quality of care, such as the NH culture-change movement and clinical practice guidelines, received increased attention. In short, LTC research trends have shifted from the demand side (care demanders) to the supply side (caregivers), and from basic studies to practical applications.

In this study, we considered references that underwent significant bursts within the past three years (2015–2017) to represent the newest LTC research frontiers. Among 16 burst references in Figure 6, some recent bursts, such as references No. 4, No. 14, No. 15, and No. 16 were identified; bursts of these four references are expected to continue to lengthen in the future and may be deserving of more attention, because bursts are often followed by subsequent increasing trends. Among 10 clusters, cluster #9 with mean publication year 2011 emerged as the newest research frontier. Moreover, to analyze the research frontiers more accurately, other references with strong recent bursts, shown in Table 4, were also identified as predictive indicators, such as references No. 9, No. 11, and No. 17. The new research frontiers in the LTC field can be summarized as follows: frailty in elderly people (#9, No. 4, No. 16, and No. 17), the NH culture-change movement (No.9 and No. 14), dementia care (No. 11), and LTC workforce and LTC financing (No. 15).

#### 3.3.2. Keyword Co-Occurrence Analysis

The main features of the intellectual landscape of LTC keywords were three-fold: high-frequency keywords; keywords with high centrality; and keywords with strong citation bursts (i.e., burst keywords). Specifically, we conjectured the hot topics by extracting high-frequency keywords [29,33]. Keywords with high centrality represent major intellectual turning points connecting other keywords, while burst keywords represent new research frontiers [25,27,30].

**Figure 7:**
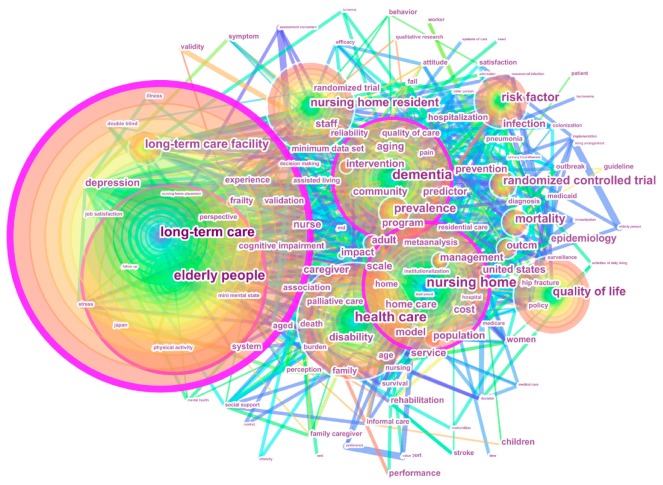
Network of co-occurring keywords.

To illustrate the hotspots in LTC research, we conducted a keywords co-occurrence analysis. The parameters in CiteSpace were remained the same except node type being changed from “Cited Reference” to “Keyword”. Keywords used in our analysis included “Author Keywords” which were supplied by authors, and “Keywords Plus” which were supplied by the journals. To avoid potential misunderstandings, some similar keywords were combined. For example, “long-term-care” and “long term care” were merged into “long-term care.” Notably, we also used a short time span (2000–2017) to construct the network of co-occurring keywords. The network consisting of 138 nodes and 841 links is shown in Figure 7. Each node represents one keyword; bigger nodes reflect higher co-occurrence frequency and each “keyword to keyword” link indicates the co-occurrence relationship.

Similarly, we identified 34 representative keywords in terms of the three features mentioned above. The top 17 keywords with a co-occurrence frequency over 550 are listed in Table 5, from No. 1 to No. 17. Five keywords from No. 1 to No. 6 had high centrality, except for keyword No. 4. Additionally, 17 keywords with the strongest burst in the group of keywords that started to burst at the same time are also summarized in Table 5, from No. 18 to No. 34. Notably, there were no burst keywords in 2004 and three burst keywords starting from 2015.

The top two keywords in terms of co-occurrence frequency were “long-term care” (4837) and “elderly people” (2351). Accordingly, elderly people were the main subjects of LTC research. Keywords with high centrality were observed in “long-term care,” “elderly people,” “nursing home,” “dementia,” and “nursing home resident,” which represented major intellectual turning points linking different keywords with significantly influenced LTC research development.

As for these 17 high-frequency keywords, according to previous scientometric studies, they can be directly regarded as LTC research hotspots. However, we believe that the hot topics identified in this way are too broad and macro to focus clearly on the major issues. To address the gap, we identified the hot research topics by integrating 17 high-frequency keywords and considering the co-occurring keywords shown in Figure 7. The resulting five main hotspots were as follows:

1. Dementia care was extracted using four keywords “long-term care,” “elderly people,” “dementia,” and “long-term care facility.” With an increasing number of people suffering from dementia, considerable attention is currently focused on improving quality of life for people with dementia [66]. Patients and caregivers are the central stakeholders in dementia care and there has been much research on the health status and care needs of elderly patients with dementia [13,14,67,68]. In terms of dementia caregivers, especially informal caregivers, compelling evidence has suggested that they suffer a high level of care burden [40,66]. Some care models for people with dementia have received increased attention, such as the ABLE model [67], person-centered care model [68], and personhood model [69].
2. Quality of care was identified using six keywords “long-term care,” “nursing home,” “health care,” “quality of life,” “quality,” and “outcomes.” The quality of care for NH residents has been a major concern internationally. Despite great improvements in NHs, LTC quality in the context of population ageing remains challenging. Within this theme, three major aspects were addressed: (1) the indicators for measuring quality of care, such as depression symptoms, psychotropic drug use, physical restraints, pain management, pressure ulcers, fall incidents, and mortality rate [53,56]; (2) factors influencing quality of care, such as nurse-staffing levels, NH profit status, and the education level and training of nursing staff [40,56]; and (3) the strategies for improving quality of care, such as implementing health information technology, enhancing quality monitoring systems, strengthening the caregiving workforce, and implementing culture-change practices in NHs [5,19,21].
3. Prevalence and risk factors were extracted using the keywords “prevalence,” “risk factor,” and “infection.” Psychiatric disorders are common among elderly people. In an effort to better understand and treat the diseases, some researchers have investigated the prevalence of and risk factors for common psychiatric disorders in LTC facilities, such as dementia, depression, bipolar disorder, anxiety disorders, schizophrenia, and alcohol use disorders [14]. Moreover, much evidence indicates a marked increase in the incidence of healthcare-associated infections caused by multidrug-resistant organisms (MDROs). Hence, prevention and control of MDROs has become a public health priority. In recent years, considerable attention has been paid to determining the prevalence of and risk factors for MDROs, such as methicillin-resistant staphylococcus aureus (MRSA), multidrug-resistant gram-negative bacilli (GNB), and vancomycin-resistant enterococci (VRE) [70].
4. Mortality comprised five representative keywords “long-term care,” “elderly people,” “mortality,” “people,” and “population.” Patient death becomes an increasingly common occurrence in LTC literature. Identifying mortality and its predictors is important for implementing of therapeutic management for high-risk patients, with the goal of improving survival. In an LTC context, the focus of this theme can be summarized into two main aspects: (1) mortality trends and differences between different groups, such as mortality of residents with regard to age, gender, and distribution of care levels under home-based or institutional care [71]; and (2) the factors associated with mortality, such as demographic characteristics, functional and cognitive status, specific diseases (cancer and heart disease), antipsychotic drug use, social support, and influenza vaccination of health-care workers [34,72].
5. Randomized controlled trial was extracted using one keyword “randomized controlled trial.” As a form of scientific experiment, it aims to evaluate the effectiveness of various types of medical interventions [34,36]. For example, one such trial was designed to determine whether vaccination of health-care workers can lower mortality and the frequency of laboratory-proven influenza infection in elderly patients in long-term care hospitals [34].

**Figure 8:**
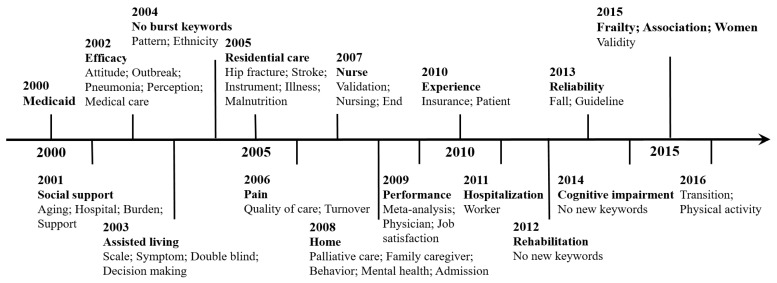
Timeline of the 17 burst keywords and 41 new keywords.

Moreover, 17 burst keywords in Table 5 were considered as indicators of research frontiers. Figure 8 shows their evolution paths. To analyze the trends more accurately, some new keywords from each year were also listed in the figure because their publication time can reflect the research evolution trends [29]. Forty-one new keywords from each year were identified. In terms of the time evolution of these keywords, LTC research trends can be categorized into three phases: in the first phase (2000–2005), the keywords reflected the topics on resident-related health or functional status and care demand; in the second phase (2006–2010), the focus shifts to caregiving-related workforce, such as keywords “nurse,” “nursing,” and “family caregiver;” and in the last phase (2011–2016), the keywords indicated an increased focus on practical exploration of the LTC field.

The keywords showing occurrence bursts within the past three years (2015–2017) were cognitive impairment (16.94, 2014–2017), frailty (46.40, 2015–2017), association (36.48, 2015–2017), and women (9.18, 2015–2017); these findings indicated that a much research attention was directed to these areas. Thus, the emerging LTC research frontiers were summarized as follows: cognitive impairment, frailty in elderly people, association analysis, and LTC for older women.

## 4. Conclusions

To the best of our knowledge, this study is the first scientometric analysis of global LTC research over the past decades. The overall investigation included: (1) the scientific output and citations of LTC research were depicted to examine its current status and development; (2) the contributions of major countries/regions, institutions, and authors were identified by visualizing collaboration networks; and (3) the hot topics, new frontiers, and evolution trends in LTC research were explored through integrating document co-citation analysis and keyword co-occurrence analysis. The remarkable findings were as follows:

A noticeable upward trend in LTC research publications over time suggested that increasing attention has been directed toward LTC. The number of publications was estimated to reach 1217 in 2018 and 1302 in 2019, confirming that LTC research is likely to remain active in the next few years. The polynomial trendline of citations indicated an inverse U-shaped relationship, especially in the period 1991–2018.

LTC research scientific publications primarily originated from the US, Canada, and England, and these three contributors formed a leading research group. Interestingly, we found that the collaborative relationships among them were not strong. To our minds, their weak collaboration might result from the reason from different LTC systems. Moreover, developing countries were still under-represented in the global research network. The top 15 most productive institutions all came from North America and Europe, confirming the importance of their contributions to LTC. Harvard University was the most powerful institution because it received high publication counts and high centrality simultaneously. Meanwhile, we noted that institutions from Asian countries have accelerated their efforts to participate in LTC research. Mor and Zimmerman were the most productive and high-impact authors, although there was no direct collaboration between them. Grabowski and Ribbe played an important role in bridging the collaborative gap between Mor and Zimmerman.

The results of visualizing the intellectual landscape of references were consistent with those of visualizing the intellectual landscape of keywords. Simultaneous consideration of both revealed that the common LTC research hot topics in the 2000–2017 period were dementia care, quality of care, prevalence and risk factors, mortality, and randomized controlled trial. The LTC evolution trends showed three stages: an early stage (2000–2005), where research was primarily focused on functional, cognitive and health status, and care demand among older LTC residents; a second stage (2006–2010), where the focus shifted to caregiving-related workforce factors, such as nurse turnover, nurse training, and home care staff vaccinations; and a third stage (2011–2015), where several practical explorations aimed at improving quality of care and clinical practice guidelines received considerable attention, such as the NH culture-change movement. This reflects a shift in LTC research trends from the demand side (care demanders) to the supply side (caregivers), and from basic studies to practical applications. In summary, LTC research has become substantially broader and deeper. The common research frontiers were frailty in elderly people and dementia care (closely related to cognitive impairment).

These results provide valuable information to LTC researchers and practitioners. A variety of visualized networks offer an in-depth understanding of the major countries/regions, institutions, researchers, hot topics, evolution trends, and new research frontiers. Moreover, for LTC practitioners, this study presents accurate information regarding the key authors and institutions best suited to assist in developing LTC policies. This scientometric review method can also be used to visualize the status and trends of other research topics. However, there was also a limitation in terms of our scientometric analysis. The retrieval strategy, i.e., treating “long-term care” as the only search term, still has room to be improved. While this study is a good starting point for reviewing the literature on LTC, for researchers who want to deeply delve into the field, they are suggested using search terms other than “long-term care” and connecting them with a Boolean operation to LTC.

## Figures and Tables

**Table 1:** Top 15 countries/regions in terms of publications and centrality.

Rank	Country/Region	Count	Centrality	Rank	Country/Region	Centrality	Count
1	USA	6308	0.07	1	England	0.16	815
2	Canada	1714	0.03	2	Denmark	0.15	35
3	England	815	0.16	3	France	0.12	380
4	Germany	650	0.02	4	Scotland	0.12	91
5	Japan	635	0.01	5	Sweden	0.11	323
6	Netherlands	628	0.02	6	Switzerland	0.10	180
7	Australia	517	0.05	7	USA	0.07	6308
8	France	380	0.12	8	Spain	0.07	312
9	Taiwan	372	0.01	9	Czech Republic	0.07	55
10	Italy	353	0.03	10	India	0.07	16
11	Sweden	323	0.11	11	Singapore	0.06	80
12	Spain	312	0.07	12	Portugal	0.06	40
13	China	250	0.05	13	Mexico	0.06	26
14	Israel	219	0.01	14	Australia	0.05	517
15	South Korea	207	0.01	15	China	0.05	250

**Table 2:** Top 15 institutions in terms of publications.

Rank	Institution	Count	Centrality	Country
1	University of Toronto (UT)	406	0.07	Canada
2	University of North Carolina (UNC)	233	0.08	USA
3	Harvard University (Harvard)	231	0.14	USA
4	University of Michigan (UM)	222	0.12	USA
5	McMaster University (McMaster)	222	0.08	Canada
6	Duke University (Duke)	219	0.03	USA
7	Brown University (Brown)	205	0.09	USA
8	University of Maryland (UMD)	197	0.06	USA
9	University of California, San Francisco (UCSF)	188	0.07	USA
10	University of Minnesota (UMN)	173	0.04	USA
11	University of Alberta (UA)	173	0.03	Canada
12	Vrije Universiteit Amsterdam (VU)	155	0.02	Netherlands
13	University of Pittsburgh (PITT)	151	0.07	USA
14	University of British Columbia (UBC)	138	0.03	Canada
15	University of Waterloo (UW)	134	0.02	Canada

**Table 3:** Top six most cited articles from Mor and Zimmerman.

Author	Title of Articles	Year	Count
Mor, V (98, Brown University)	Randomised trial of impact of model of integrated care and case management for older people living in the community	1998	242
	Driven to tiers: Socioeconomic and racial disparities in the quality of nursing home care	2004	187
	Does receipt of hospice care in nursing homes improve the management of pain at the end of life?	2002	157
	Validity of diagnostic and drug data in standardized nursing home resident assessments-Potential for geriatric pharmacoepidemiology	1998	141
	The OBRA-87 nursing home regulations and implementation of the resident assessment instrument: Effects on process quality	1997	141
	A comprehensive clinical assessment tool to inform policy and practice: Applications of the Minimum Data Set	2004	121
Zimmerman, S (89, University of North Carolina (UNC) at Chapel Hill)	Nursing home facility risk factors for infection and hospitalization: Importance of registered nurse turnover, administration, and social factors	2002	139
	Attitudes, stress, and satisfaction of staff who care for residents with dementia	2005	125
	Assisted living and nursing home: Apples and oranges?	2003	121
	Dementia care and quality of life in assisted living and nursing homes	2005	117
	Evaluating the quality of life of long-term care residents with dementia	2005	103
	High-intensity environmental light in dementia: Effect on sleep and activity	2007	74

**Table 4:** Forty representative references in terms of citations, centrality, and bursts.

No.	Count	Centrality	Strength	Reference	Year	Begin	End	Cluster ID
1	93	0.17	28.45	Carman et al. [34]	2000	2001	2008	#7
2	74	0.01	17.96	Luppa et al. [35]	2010	2012	2017	#6
3	65	0.02	21.85	Hayward et al. [36]	2006	2008	2013	#7
4	61	0.00	25.53	Cruz-Jentoft et al. [37]	2010	2014	2017	#9
5	61	0.04	19.61	Gaugler et al. [38]	2007	2012	2015	#6
6	58	0.05	14.56	Mitchell et al. [13]	2009	2011	2015	#1
7	57	0.04	16.49	Seitz et al. [14]	2010	2012	2017	#1
8	57	0.06	14.60	Institute of Medicine [19]	2008	2010	2017	#2
9	57	0.01	23.84	Koren [39]	2010	2014	2017	#1
10	54	0.08	14.63	Tsutsui and Muramatsu [4]	2007	2009	2015	#2
11	53	0.01	22.15	WHO [40]	2012	2014	2017	#1
12	53	0.04	15.07	Ouslander et al. [41]	2010	2013	2017	#8
13	53	0.03	17.95	Kaye et al. [6]	2010	2014	2017	#6
14	50	0.06	15.45	Rahman and Schnelle [21]	2008	2011	2017	#1
15	50	0.01	17.51	Colombo et al. [5]	2011	2013	2017	#6
16	49	0.04	23.41	Morley et al. [42]	2013	2015	2017	#9
17	48	0.01	22.93	Clegg et al. [43]	2013	2015	2017	#9
18	48	0.01	16.81	Ouslander et al. [44]	2011	2013	2017	#8
19	46	0.02	16.10	Onder et al. [45]	2012	2013	2017	#1
20	45	0.05	19.65	Potter et al. [46]	1997	2000	2005	#5
21	44	0.06	14.98	Kane [10]	2001	2003	2009	#4
22	44	0.02	18.36	Bonsang [47]	2009	2014	2017	#6
23	42	0.03	18.33	Wallace et al. [48]	1998	2000	2005	#0
24	41	0.02	22.06	Hawes et al. [49]	1995	2000	2003	#3
25	40	0.05	15.12	Manton and Gu [50]	2001	2004	2009	#0
26	20	0.25	8.37	Intrator et al. [51]	2004	2007	2011	#8
27	34	0.19	12.69	Harrington et al. [52]	2000	2001	2007	#3
28	20	0.18	6.48	Mor et al. [53]	2003	2004	2010	#2
29	38	0.17	9.04	Mor et al. [54]	2010	2015	2017	#8
30	16	0.15	6.90	Nicolle [55]	2001	2005	2009	#5
31	16	0.14	6.46	Hillmer et al. [56]	2005	2009	2013	#2
32	38	0.12	17.40	Morris et al. [57]	1999	2003	2007	#4
33	28	0.12	11.92	Castle and Engberg [17]	2005	2006	2010	#2
34	26	0.12	10.93	Loeb et al. [58]	1999	2002	2007	#5
35	5	0.11	-	Lunney et al. [12]	2003	-	-	#0
36	13	0.10	5.45	Intrator et al. [59]	1999	2002	2007	#5
37	11	0.10	-	Arno et al. [60]	1999	-	-	#0
38	32	0.01	12.15	Zimmerman et al. [61]	2003	2005	2010	#4
39	27	0.02	11.31	Bowers et al. [62]	2003	2007	2011	#2
40	38	0.02	14.91	Kane et al. [63]	2007	2010	2014	#1

**Table 5:** Thirty-four representative keywords in terms of occurrences, centrality, and bursts.

No.	Count	Centrality	Keyword	No.	Strength	Begin	End	Keyword
1	4837	0.29	Long-term care	18	19.29	2000	2008	Medicaid
2	2351	0.11	Elderly people	19	10.73	2001	2008	Social support
3	2137	0.12	Nursing home	20	13.33	2002	2005	Efficacy
4	2072	0.09	Health care	21	11.47	2003	2005	Assisted living
5	1872	0.13	Dementia	22	17.95	2005	2009	Residential care
6	1647	0.10	Nursing home resident	23	15.28	2006	2010	Pain
7	1217	0.07	Risk factor	24	21.74	2007	2012	Nurse
8	983	0.09	Quality of life	25	7.37	2008	2009	Home
9	879	0.05	Prevalence	26	15.00	2009	2010	Performance
10	873	0.08	Mortality	27	10.61	2010	2015	Experience
11	861	0.07	Long-term care facility	28	9.79	2011	2012	Hospitalization
12	737	0.02	People	29	11.05	2012	2013	Rehabilitation
13	688	0.04	Randomized controlled trial	30	23.40	2013	2015	Reliability
14	643	0.04	Quality	31	16.94	2014	2017	Cognitive impairment
15	617	0.05	Management	32	46.40	2015	2017	Frailty
16	568	0.02	Outcomes	33	36.48	2015	2017	Association
17	561	0.02	Population	34	9.18	2015	2017	Women
